# Draft Genome Sequences of Streptomyces sp. Strains MH60 and 111WW2

**DOI:** 10.1128/genomeA.00356-18

**Published:** 2018-05-03

**Authors:** Louise F. Thatcher, Cindy A. Myers, Cathryn A. O’Sullivan, Margaret M. Roper

**Affiliations:** a CSIRO Agriculture and Food, Centre for Environment and Life Sciences, Wembley, Western Australia, Australia

## Abstract

We report here the draft genome sequences, annotations, and predictions of secondary metabolite gene clusters of two endophytic Streptomyces species isolated from wheat plants growing in the Western Australian wheat belt. These strains, Streptomyces sp. strains MH60 and 111WW2, possess antifungal and/or plant growth-promoting activities.

## GENOME ANNOUNCEMENT

Actinobacteria are known to exhibit antibiotic properties against other microbes and to promote plant growth, with many species of the genus Streptomyces being endophytes (1, 2). We isolated endophytic actinobacteria from the roots of healthy wheat plants growing in areas of fields known to consistently perform well in terms of plant health and grain yield. Plant roots were surface sterilized, aseptically cut, and plated on agar medium selective for actinobacteria (3). The isolates were screened for the suppression of plant-pathogenic fungal growth in *in vitro* competition tests. Streptomyces sp. strains MH60 and 111WW2 were capable of suppressing a range of fungal pathogens, including Rhizoctonia solani, Fusarium pseudograminearum, Pythium spp., Gaeumannomyces graminis var. tritici, and Sclerotinia sclerotiorum to various degrees of efficacy (3, 4). Sequencing of 16S rRNA designated the above-mentioned strains Streptomyces species (4).

DNA for whole-genome sequencing was extracted from mycelia and spores using a MiBio PowerLyzer UltraClean microbial DNA isolation kit. Indexed Illumina TruSeq libraries (350-bp inserts) were prepared by the Australian Genome Research Facility (AGRF), Melbourne, Australia, and sequenced using 150-bp paired-end reads on an Illumina MiSeq instrument, using approximately 2/10 of a sequencing lane. A total of 0.44 and 0.45 Gbp of raw data were generated from this sequence run for MH60 and 111WW2, respectively. Reads were trimmed using cutadapt (5) and sorted as per Thatcher et al. (6), and overlapping reads merged using FLASH (version 1.2.11) (7). Reads (paired-end, singletons, and merged) were assembled *de novo* using SPAdes (version 3.9.0) (8) with the “–careful” option and k-mer lengths of 21, 33, 55, and 77. Contigs less than 1,000 bp were removed. The strain MH60 genome was assembled into 8.14 Mbp (190 scaffolds; *N*_50_, 27 scaffolds), and the 111WW2 genome assembled into 8.59 Mbp (236 scaffolds; *N*_50_, 41 scaffolds). Both genomes had a G+C content of 72%. Coding sequences, functional annotation, and secondary metabolite biosynthesis gene clusters were predicted by Prokka (version 1.11) (9) (incorporating Prodigal version 2.6.3 [10]), Blast2GO (version 1.0.2) (11), and antiSMASH (version 3.0.5.1) (12), respectively.

Blast2GO (11) best BLAST hits analysis for species comparisons revealed the nearest-neighbor species for MH60 to be Streptomyces canus and Streptomyces aureofaciens, while the nearest-neighbor species for 111WW2 were of the Streptomyces violaceoruber clade (S. lividans and S. coelicolor).

A total of 7,340 coding sequences were predicted by Prokka (9) for MH60, and 7,849 sequences were predicted for 111WW2. The prediction of secondary metabolite clusters by antiSMASH (12) suggested that their genomes harbor 26 biosynthetic gene clusters each, including those coding for polyketide synthases, nonribosomal peptide synthetases, and others, such as bacteriocin, siderophore, or ectoine clusters, suggesting their potential to produce diverse secondary metabolites and antimicrobial peptides.

### Accession number(s).

These whole-genome shotgun projects for Streptomyces sp. strains MH60 and 111WW2 have been deposited at DDBJ/ENA/GenBank under the accession numbers MULI00000000 and MUYY00000000, respectively, and the corresponding versions described in this paper are the first versions, MULI01000000 and MUYY01000000.
